# A Case of Type 2 Hypersensitivity to Rasburicase Diagnosed with a Natural Killer Cell Activation Assay

**DOI:** 10.3389/fimmu.2018.00110

**Published:** 2018-01-29

**Authors:** Sébastien Viel, Rémi Pescarmona, Alexandre Belot, Audrey Nosbaum, Christine Lombard, Thierry Walzer, Frédéric Bérard

**Affiliations:** ^1^Laboratoire d’Immunologie, Centre Hospitalier Lyon Sud, Hospices Civils de Lyon, Lyon, France; ^2^Centre International de Recherche en Infectiologie, CIRI, INSERM U1111, CNRS UMR5308, Ecole Normale supérieure de Lyon, Université Claude Bernard Lyon I, Lyon, France; ^3^Service de Néphrologie, Rhumatologie, Dermatologie pédiatriques, CNR RAISE, Hôpital Femme Mère Enfant, Hospices Civils de Lyon, Bron, France; ^4^Service d’Immunologie Clinique et d’Allergologie, Centre Hospitalier Lyon Sud, Hospices Civils de Lyon, Lyon, France

**Keywords:** rasburicase, type 2 hypersensitivity, natural killer cells, ADCC, antibody dependent cell cytotoxicity

## Abstract

Drug hypersensitivity reactions can lead to different clinical pictures depending on the underlying immunological mechanism. Diagnosis tests are already available to assess the most frequent drugs hypersensitivity reactions, which are mediated by specific IgE or T cells. However, it remains challenging to diagnose type 2 hypersensitivity reactions (T2HR), which can lead to severe cytopenia and liver failure. Here, we describe a case of T2HR to rasburicase, an uricolytic agent used to prevent tumor lysis syndrome. In this patient, sensitization was associated with the production of specific IgG able to bind to leukocytes. We found that patient NK cells were specifically activated in the presence of rasburicase and autologous serum, which led to exocytosis of lytic granules. This antibody-dependent cell cytotoxicity mechanism may lead to cytopenia observed in the patient. Moreover, this NK cell activation assay could be used to improve the diagnosis of a T2HR to rasburicase and, by extent, to other drugs. These data also suggest that NK cells could play an important role in the pathophysiological mechanism of T2HR.

Type 2 hypersensitivity reactions (T2HR) are antigen-specific semi-delayed reactions according to Gell and Coombs classification (1). These reactions involve IgM/IgG-mediated cytotoxicity of various hematopoietic and non-hematopoietic cells. Our current understanding of the underlying mechanism is that specific immunoglobulins are generated against allergens bound to membrane proteins during the sensitization phase and mediate the reaction upon challenge with the same allergen. Clinical symptoms depend on the target cell lineage and appear within hours after a second contact with the antigen. They are caused by the elimination of antibody-coated cells by antibody-dependent cell-mediated cytotoxicity (ADCC), antibody-dependent cell-mediated phagocytosis (ADCP), or complement activation.

Many drugs are considered as haptens that require conjugation with a carrier protein to become immunogenic (2). Theoretically, drugs can cause the four types of hypersensitivity reactions described by Gell and Coombs (3). The most frequent drug hypersensitivity reactions are mediated by IgE (type 1) or by specific T cells (type 4) for which clinical (4) and biological diagnosis tests (5) are available. Drugs can also induce type 2 hypersensitivity reactions that can lead to anemia, thrombopenia, neutropenia, or hepatitis. These reactions can only be diagnosed accurately using the drug provocation test (DPT), since skin tests are not reliable and no biological tests are currently available. However, DPT represents a high-risk method of diagnosis testing, as it can reproduce the type 2 hypersensitivity reaction.

Natural killer (NK) cells are innate lymphocytes involved in the defense against intracellular pathogens and tumors (6). They have the ability to kill other cells recognized as targets through an arsenal of receptors recognizing major histocompatibility complex (MHC) class I molecules or various surface ligands associated with cellular stress. They are also able to detect and kill IgG coated cells *via* CD16/FcγRIIIA, an activating receptor that drives ADCC in NK cells (6). The main cytotoxic pathway involves the release of cytotoxic proteins such as perforin, granzymes, and granulysin. NK cells also secrete large amounts of IFN-γ and other cytokines in response to stimulation. Numerous *in vitro* tests using either anti-CD16-coated plates, or monoclonal antibody-coated target cells are available to monitor ADCC function of NK cells in human.

Here, we describe the case of a type 2 reaction to rasburicase, a potent uricolytic agent, reducing uric acid level and preventing its accumulation in patients with hematologic malignancies or at high risk of tumor lysis syndrome. Due to its protein nature and its absence in humans, rasburicase is considered as highly immunogenic leading to a sensitization rate of about 5% (7). However, only immediate hypersensitivity reactions were reported and this drug was never associated with T2HR to our knowledge. We applied an NK cell assay to diagnose this reaction, and we propose that such tests could be broadly used for the diagnosis of T2HR.

A 62-year-old male patient presented fever (39°C) and tachycardia (120 beats/minute) during his fourth chemotherapy course associating pentostatine, rasburicase, and allopurinol to treat a tricholeukocyte leukemia. The day after, the fever persisted and the patient developed thrombopenia, lymphopenia, and liver failure (Table 1). Then, a sepsis state occurred, and the patient was transferred to intensive care unit (ICU) and treated with piperacillin/tazobactam and amikacin. His condition rapidly improved. Two months later, a drug allergy work up was performed in order to investigate the nature of the previous episode. Pricks tests with rasburicase were negative and the patient received rasburicase as DPT (5.5 mg). One hour after the injection, the patient presented a new fever episode (39.5°C). Two hours later, a liver failure associated with lymphopenia and thrombopenia relapsed (Table 1) and the patient was transferred to ICU for the second time.

**Table 1:** Clinical and biological characteristics of the patient who developed a semi-delayed reaction to rasburicase.

	Episode 1		Episode 2			
Delay	Day +1	Day +2	Before rasburicase	3 h	6 h	Day +1
Fever	Yes	Yes	No	Yes	Yes	No
Leukocytes (4–10 G/L)	17.5	8.7	4.3	1.3	2.5	9.4
Neutrophils (1.8–7 G/L)	17.3	8.3	2.5	1.21	1.2	8.9
Lymphocytes (1–4 G/L)	0.2	0.2	1.4	0.05	0.03	0.2
Platelets (150–400 G/L)	92	55	125	98	85	81
GOT (0–34 UI/L)	773	461	26	86	318	122
GPT (0–55 UI/L)	1,162	863	43	73	257	220
LDH (125–220 UI/L)	1,339	828	180	247	446	NR
γGT (12–64 UI/L)	307	225	22	99	202	171
Total bilirubin (3–20 μmol/L)	56	101	7	17	26	NR
Conjugated bilirubin (0–10 μmol/L)	46	85	NR	NR	14	NR
PR (75–100%)	49	49	98	90	77	54
Factor V (60–120%)	61	NR	NR	NR	NR	56

*GOT, glutamic oxaloacetic transaminase; GPT, glutamic pyruvic transaminase; LDH, lactate dehydrogenase; γGT, gamma-glutamyl transferase; PR, prothrombin ratio; (), normal values; NR, not realized*.

To study the immunological mechanism of this drug reaction, we first tested if the patient was sensitized to the drug. Once the patient gave his written informed consent (in accordance with the Declaration of Helsinki), his serum was incubated with his own leukocytes in the presence of the drugs he received during his first fever episode [rasburicase (Sanofi, Paris, France) or allopurinol (HAC Pharma, Caen, France) at 100 mg/L final]. Then, cells were stained with an antihuman IgG coupled to Phycoerythrin and the Mean Fluorescence Intensity (MFI) was measured by flow cytometry. We observed that the incubation of patient serum with rasburicase leads to a specific IgG staining on monocytes and lymphocytes but not on neutrophils, while IgG MFI was not increased in the presence of allopurinol (Figure 1A). This phenomenon was also observed, albeit at a lower extent, when patient serum was incubated with rasburicase and leukocytes from a healthy donor (Figure 1B).

**Figure 1:**
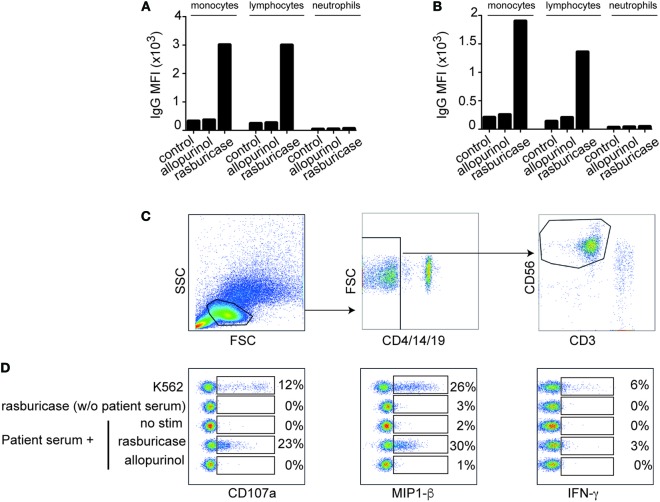
Mean fluorescence intensity of anti-IgG coupled to Phycoerythrin staining after incubation of patient serum with the indicated drug on monocytes, lymphocytes, and neutrophils from patient **(A)** or a healthy control **(B)**. **(C)** Gating strategy allowing the identification of natural killer (NK) cells (CD3^−^/4^−^/14^−^/19^−^/56^+^ cells). **(D)** Concatenation of FACS plot showing NK cells activation markers after NK cells culture *in vitro* in the indicated conditions. Results are expressed in percentage of positive cells for each condition.

Next, we performed an NK-cell activation assay to study if these coated IgGs were functional and able to activate NK cells *in vitro* through an ADCC mechanism. Briefly, patient PBMCs were incubated 4 h in complete medium with or without 10% of patient serum in the presence or absence of the drugs he received during his first episode [rasburicase (Sanofi, Paris, France) and allopurinol (HAC Pharma, Caen, France) at 100 mg/L final] or with NK-sensitive targets K562 cells (8) at a 1:1 ratio (positive control). Golgi-stop (BD Biosciences, Franklin Lakes, NJ, USA) was added after 1 h of culture. Cells were then stained for CD3, CD4, CD19, CD56 (Beckman Coulter, Brea, CA, USA), and CD107a (eBioscience, Paris, France) surface expression. IFN-γ (eBioscience, Paris, France) and MIP-1β (BD Biosciences, Franklin Lakes, NJ, USA) intracellular stainings were then performed after cell permeabilization using Cytofix/Cytoperm (BD Biosciences, Franklin Lakes, NJ, USA). Tubes were run on a Beckman-Coulter Navios instrument (Beckman Coulter, Brea, CA, USA), and data were analyzed with FlowJo (Treestar, Ashland, OR, USA). NK cells were defined as CD3^−^/4^−^/19^−^CD56^+^ cells as shown in Figure 1C.

Natural killer cells’ activation was observed when autologous PBMCs were incubated with rasburicase in the presence of the patient’s serum. Under these conditions, NK cells degranulated (increase of the CD107a expression) and produced high amount of MIP-1β similar to positive control with K562 cells (Figure 1D), whereas the production of IFN-γ was low in all conditions. By contrast, when PBMCs were incubated with rasburicase without patient serum [but with 10% fetal calf serum (FCS)] or with patient serum but without any drug or with allopurinol, no sign of NK cells activation was observed (Figure 1D). These results suggest that NK cells’ activation requires the presence of specific immunoglobulins present in the patient’s serum that recognize rasburicase complexed to a cell surface antigen. Since this episode, the patient is in complete remission of his hemopathy.

Rasburicase is a recombinant form of urate oxidase, which reportedly causes less allergic reactions than its previously non-recombinant form (Uricozyme) (9). For drugs, functional *in vitro* biological tests to confirm the type 2 nature of a drug hypersensitivity are missing and diagnosis is usually made on the basis of clinical symptoms. Other studies are needed to define the sensitivity/specificity of this NK cells test, but according to us, this assay could limit the realization of DPT and its risks in case of suspicion of a type 2 drug hypersensitivity. Whether NK cells participate to the allergic reaction *in vivo* remains to be determined. In addition to NK cells, CD16 is expressed on a wide variety of immune cells, including neutrophils, monocytes, and macrophages. Mouse studies clearly established that the mechanism of cytotoxic action of IgGs is largely dependent on myeloid cells and in particular resident macrophage populations (10). The present test based on NK cells could be of interest to fairly diagnose drug type 2 hypersensitivity to rasburicase and also to other drugs, by extent.

## Ethics Statement

The patient of this case report gave his written informed consent (in accordance with the Declaration of Helsinki).

## Author Contributions

SV has performed experiments, drew the figure, and wrote the manuscript. RP has compiled clinical data and drew the table. AB, AN, and CL have participated in the design of the experiments and wrote the manuscript. FB was in charge of the patient care and wrote the manuscript. TW participated in the design of the experiments and the data analysis and wrote the manuscript.

## Conflict of Interest Statement

The authors declare that the research was conducted in the absence of any commercial or financial relationships that could be construed as a potential conflict of interest.
